# Dietary Oxalate Loading Impacts Monocyte Metabolism and Inflammatory Signaling in Humans

**DOI:** 10.3389/fimmu.2021.617508

**Published:** 2021-02-25

**Authors:** Parveen Kumar, Mikita Patel, Robert A. Oster, Vidhush Yarlagadda, Adam Ambrosetti, Dean G. Assimos, Tanecia Mitchell

**Affiliations:** ^1^ Department of Urology, University of Alabama at Birmingham, Birmingham, AL, United States; ^2^ Department of Medicine, University of Alabama at Birmingham, Birmingham, AL, United States

**Keywords:** monocytes, kidney stones, oxalate, inflammation, metabolism, mitochondria

## Abstract

Diet has been associated with several metabolic diseases and may impact immunity. Increased consumption of meals with high oxalate content may stimulate urinary calcium oxalate (CaOx) crystals, which are precursors to CaOx kidney stones. We previously reported that CaOx stone formers have decreased monocyte cellular bioenergetics compared to healthy participants and oxalate reduces monocyte metabolism and redox status *in vitro*. The purpose of this study was to investigate whether dietary oxalate loading impacts monocyte cellular bioenergetics, mitochondrial complex activity, and inflammatory signaling in humans. Healthy participants (n = 40; 31.1 ± 1.3 years) with a BMI of 24.9 ± 0.6 kg/m^2^ consumed a controlled low oxalate diet for 3 days before drinking a blended preparation of fruits and vegetables containing a large amount of oxalate. Blood and urine were collected before (pre-oxalate) and for 5 h after the oxalate load to assess urinary oxalate levels, monocyte cellular bioenergetics and mitochondrial complex activity, and plasma cytokine/chemokine levels. Urinary oxalate levels significantly increased in post-oxalate samples compared to pre-oxalate samples. Monocyte cellular bioenergetics, mitochondrial complex I activity, and plasma cytokine and chemokine levels were altered to varying degrees within the study cohort. We demonstrate for the first time that dietary oxalate loading may impact monocyte metabolism and immune response in a cohort of healthy adults, but these response are variable. Further studies are warranted to understand oxalate mediated mechanisms on circulating monocytes and how this potentially influences CaOx kidney stone formation.

**Clinical Trial Registration:**

ClinicalTrials.gov, identifier NCT03877276.

## Introduction

Kidney stone formation is associated with several systemic disorders which may be linked to diet including obesity, chronic kidney disease (CKD), diabetes, and cardiovascular disease (1–5). Oxidative stress and inflammation can alter cellular function and immune responses and this may play a role in these disorders (6–8). Certain dietary components have been reported to influence oxidative stress and immunity (9–11). Meals containing large amounts of oxalate can increase urinary oxalate excretion (12, 13), which is a risk factor for the development of calcium oxalate (CaOx) kidney stones as well as CKD progression (3, 14).

Oxalate is a small molecule found in plant derived foods such as spinach and nuts (14). It is absorbed in the gastrointestinal tract and excreted in urine (15). A transient increase in urinary oxalate excretion can develop 2–6 h after oxalate ingestion (13, 16, 17). During this interval, urine can become super-saturated with stone forming salts leading to the generation of crystals which may eventuate in kidney stone formation. Consumption of meals rich in oxalate augments urinary oxalate excretion potentially leading to new kidney stone formation, growth of existing stones, and in some instances renal damage (12). Experimental studies have shown renal epithelial cells exposed to oxalate have reduced glutathione levels (18), impaired DNA synthesis (19, 20), and increased reactive oxygen species (ROS) generation (18, 20–23), mitochondrial permeability transition-mediated cell death (24), and monocyte chemoattractant protein (MCP-1) secretion (25, 26). MCP-1 is a cytokine that recruits monocytes and macrophages to tissues to remove dead cells, pathogens, and crystals (27).

Mitochondria are important for monocyte health and impaired mitochondrial function can cause cellular injury and alter immune response (28, 29). We previously reported that CaOx stone formers have decreased monocyte cellular bioenergetics compared to healthy participants (30). We further showed oxalate suppresses cellular bioenergetics and disrupts redox homeostasis in a human monocytic cell line and primary monocytes from healthy adults (31). This is important as this may impact the ability of monocytes to differentiate into macrophages, the latter which are involved in crystal removal. It is not known whether dietary oxalate can alter monocyte metabolism and immunity in humans. The purpose of this study was to identify whether a large dietary oxalate load alters monocyte cellular bioenergetics, mitochondrial complex activity, and inflammatory signaling in healthy adults. We determined that dietary oxalate loading increases urinary oxalate excretion, induces nanocrystalluria, and alters monocyte metabolism and immune responses in healthy participants. Findings from this study suggest that dietary oxalate loading may influence cellular bioenergetics and immunity which could potentially lead to kidney stone formation.

## Materials and Methods

### Reagents

Anti-CD14^+^ antibody was purchased from Miltenyi Biotec Inc. (San Diego, CA). Oligomycin, FCCP [carbonyl cyanide 4-(trifluoromethoxy) phenylhydrazone], antimycin A, ADP, pyruvate, malate, rotenone, succinate, ascorbate, TMPD (N,N,N′,N′-Tetramethyl-p-phenylenediamine), and azide were purchased from Sigma-Aldrich (St. Louis, MO). The XF Plasma Membrane Permeabilizer reagent was from Agilent Technologies (Santa Clara, CA).

### Study Approval

This study adhered to the Declaration of Helsinki, was approved by the University of Alabama at Birmingham Institutional Review Board, and is registered on clinicaltrials.gov (NCT03877276). Written informed consent was obtained from all study participants.

### Study Enrollment

**Figure 1:**
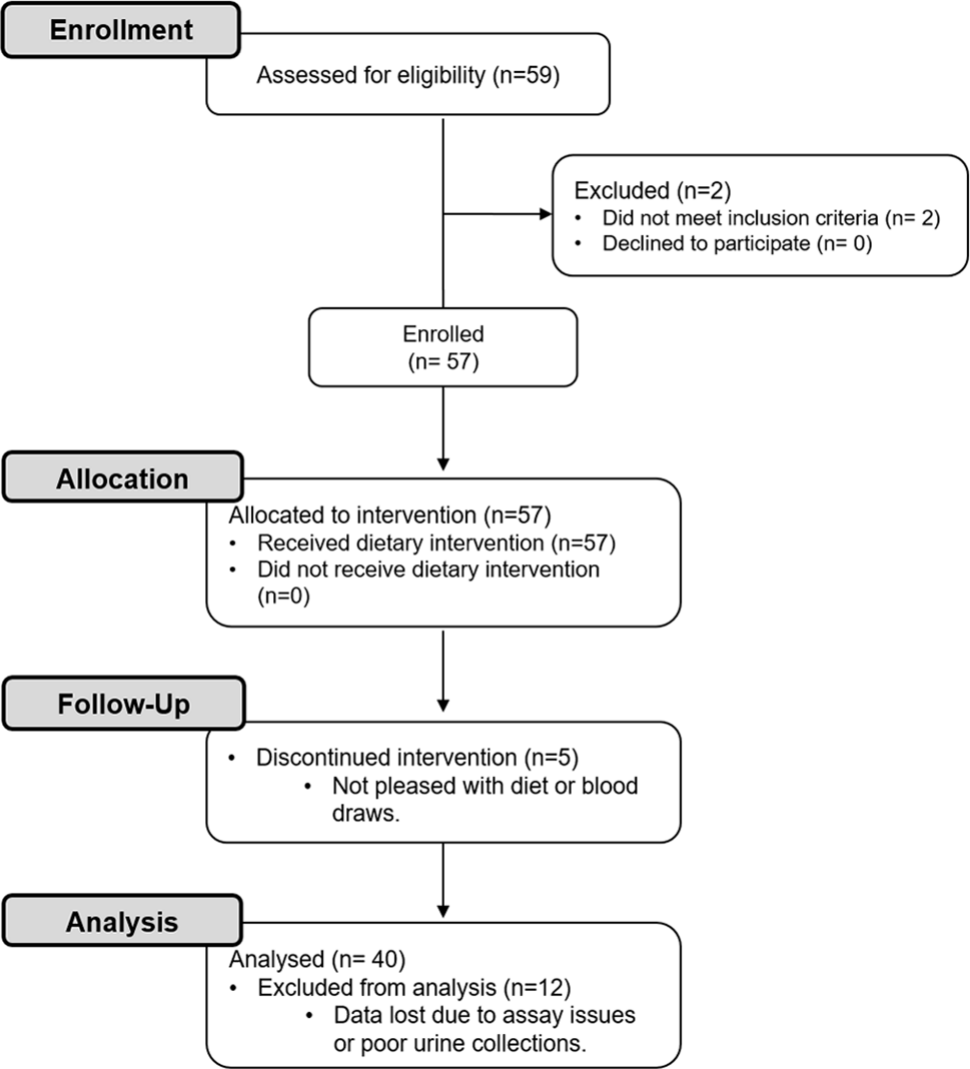
CONSORT flow diagram. Healthy participants were enrolled in this study to investigate the effects of a dietary intervention [i.e., 3-day low oxalate diet followed by a blended liquid preparation of spinach (primary source of oxalate), avocado, banana, and orange juice] on urinary oxalate levels, nanocrystalluria, and monocyte cellular bioenergetics.

A flow diagram of this study is shown in **Figure 1**. Potential participants were screened with a fasting serum comprehensive metabolic profile (CMP) and/or a urine pregnancy test. Forty healthy adults with an average age of 31.1 ± 1.3 years and a BMI of 24.9 ± 0.6 kg/m^2^ were included (**Table 1**). Participants had a normal CMP (**Supplementary Table 1**), no active medical problems or a history of kidney stone disease, and were not pregnant. Participants self-identified as being either white (60%), black (23%), or Asian (18%) (**Table 1**). Of these individuals, 63% were women and 13% had a family history of kidney stone disease. All participants agreed to refrain from taking vitamins and exercising while on the study.

**Table 1:** Study Participant Demographics.

**Demographics**	
*n*	40
Age, years	31.1 ± 1.3
Female	63%
BMI, kg/m^2^	24.9 ± 0.6
**Race**	
White	60%
Black	23%
Asian	18%
**History of Kidney Stones**	0%
**Family History of Stones**	13%

BMI, body mass index.

Values are expressed as mean ± SE; % of participants with listed parameter.

### Controlled Diets and Clinical Procedures

The UAB Center for Clinical and Translational Science (CCTS) Bionutrition Core prepared all controlled diets for this study. Diets totaled 2,000 kcal per day with known amounts of oxalate (50 mg), calcium (1,000 mg), macronutrients, and micronutrients (13). Participants consumed these controlled diets for 3 days before fasting overnight. The following morning, participants voided at 5 am and drank 1 L of water before coming to the CCTS Clinical Research Unit (CRU). Upon arrival at 7 am, participants completed a timed (5–7 am) urine collection in clean plastic bottles without preservatives (2^nd^ void of the day). The nursing staff collected blood from each participant *via* venipuncture. Urine and blood were immediately processed by study staff and kept at room temperature (RT). All participants then consumed a low oxalate breakfast and a blended liquid preparation of fruits and vegetables (spinach, avocado, banana, and orange juice) containing ~8 mmoles of oxalate. A subset of these individuals also consumed the liquid preparation containing the same foods except spinach (the primary source of oxalate) on a separate occasion. The blended liquid preparations were made in large batches and frozen to reduce variability. The oxalate content of each batch was determined using ion chromatography-mass spectrometry (IC-MS). After consuming the oxalate load, participants collected and stored their urine for 5 h at RT and consumed water only. At the end of this time point, participants delivered their urine to the CRU and had their blood drawn. All blood and urine samples were processed immediately as subsequently described.

### Urine Analyses

Urine pH and volume were measured following sample collection. Urine osmolality was determined using a Wescor Vapro 5520 Vapor Pressure Osmometer (Logan, UT). Urine was centrifuged for 10 min at 4,000 RPM at RT prior to processing samples as previously described (32). In brief, the urine supernatant was collected and either non-acidified or acidified with 2N HCl. Non-acidified urine was used to assess creatinine using a Medica EasyRA chemical analyzer (Medica, Bedford, MA) and to measure citrate levels using a standard plate reader (Citrate Assay Kit; Abcam, Cambridge, UK). Acidified urine was used to measure calcium and phosphate levels using the EasyRA chemical analyzer and to assess oxalate levels using IC-MS (33, 34). The pellets collected following centrifugation were washed twice with absolute ethanol (4,000 RPM for 10 min at RT) prior to being dried with argon gas or resuspended in absolute ethanol. Dried pellets were acidified with 0.5 M HCl to evaluate crystalline oxalate concentrations using IC-MS (33). The size and concentration of nanocrystals in the resuspended pellets (<1 µM) were determined using nanoparticle tracking analysis (NTA) and a NanoSight NS300 machine (Malvern Instruments, Inc., Malvern, UK) as we have previously described (31).

### Circulating Monocyte Isolation, Cellular Bioenergetics, and Mitochondrial Complex Activity Analyses

Blood was processed to isolate non-activated monocytes as previously described (35, 36). In brief, the mononuclear fraction was collected to positively select for monocytes using anti-CD14^+^ magnetic antibody beads. Monocytes were subsequently counted using the Bio-Rad TC20 Automated Cell Counter (Bio-Rad, Hercules, CA). Cells were seeded (150,000 cells/well) on Cell-Tak coated Seahorse plates before performing a mitochondrial stress test and assessing mitochondrial Complex I, II, and IV activity using a Seahorse XF96 Analyzer (Agilent Technologies, Santa Clara, CA) (35, 37). The mitochondrial stress test was used to measure individual bioenergetic oxygen consumption rate (OCR) parameters (i.e., Basal, ATP-linked, proton leak, maximal, reserve capacity, and non-mitochondrial). Basal respiration reflects the energetic demand of the cell under normal baseline conditions. ATP-linked respiration represents the amount of OCR that is being used to generate ATP. Proton leak is the portion of basal OCR that is not coupled to ATP generation. Maximal respiration represents the cell’s highest capacity of oxygen consumption. Reserve capacity is an indicator of the cells ability to respond to energetic demand. Lastly, non-mitochondrial OCR is an assessment of the amount of respiration that occurs once the electron transport is inhibited. The assays used to assess mitochondrial complex activity consisted of permeabilizing cells and adding different substrates as previously described (37). All data were normalized to cell count.

### Plasma Cytokine Levels

A Bio-Plex Multiplex Immunoassay System was used to measure 17 cytokine and chemokine (IL-6, IL-1β, IL-2, IL-4, IL-5, IL-6, IL-7, IL-8, IL-10, IL-12, IL-13, IL-17A, MCP-1, MIP-1β, TNF-ɑ, G-CSF, GM-CSF, and IFN-γ) levels according to the manufacturer’s instructions. In brief, premixed beads were added to a 96-well plate from the kit. Plates were washed prior to adding 50 µl of standards and samples (4× dilution) to each well containing coated beads. The plate was placed on a shaker and incubated for 1 h at RT (850 RPM). Afterward, the plate was washed with wash buffer (3×) and detection antibodies were added to each well prior to incubating the plate again for 30 min on the shaker (850 RPM at RT). The plate was washed with wash buffer (3×) prior to adding streptavidin-PE (SA-PE) for 10 min. The plate was washed with wash buffer (3×) prior to measuring samples using the BioPlex 200 machine in the UAB Comprehensive Flow Cytometry Core. Data were analyzed using the BioPlex 200 platform.

### Statistical Analyses

Descriptive statistics including means and standard errors (SEM) were calculated for study variables. Continuous variables were determined to follow at least an approximate normal distribution based on box plots, stem-and-leaf plots, and normal probability plots. Comparisons of pre-to-post changes were performed using the paired t-test. Unadjusted correlations between pairs of parameters were obtained through the use of Pearson correlation analysis. Multiple linear regression analyses were performed with each of the monocyte cellular bioenergetic responses and urinary analyte levels serving as the dependent variable and then as the independent predictor variable. Each of the regression models were adjusted for age and gender. A sensitivity analysis was then performed by running the same statistical analyses using the first group of 20 participants and then the second group of 20 participants (these groups are described previously); the results obtained for each group of 20 are similar to those obtained for the entire group of 40. Statistical tests were two-sided and performed at a significance level of 5%. Statistical analyses were performed using SAS (version 9.4; SAS Institute, Cary, NC). All graphs were made using GraphPad Prism (version 7; La Jolla, CA).

## Results

### Dietary Oxalate Loading Alters Urinary Analyte Excretion and Induces Nanocrystalluria

**Figure 2:**
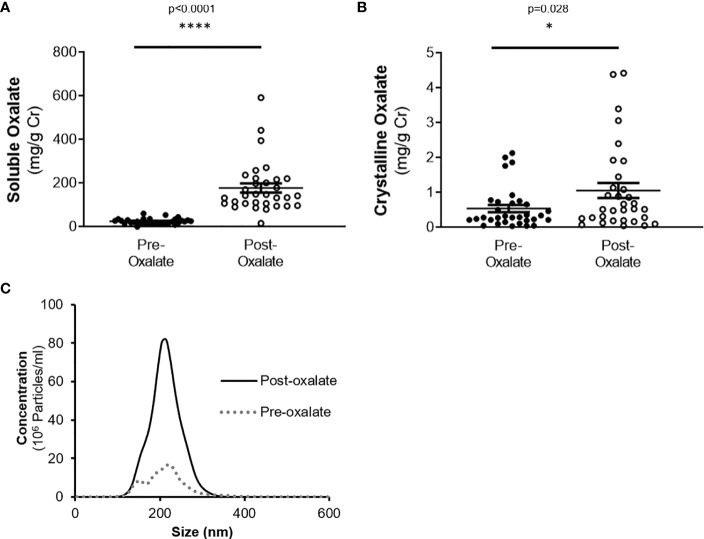
Urinary oxalate levels and nanocrystalluria concentrations are increased in response to a dietary oxalate load. **(A)** Soluble and **(B)** crystalline urinary oxalate levels in healthy participants. **(C)** Representative profile of crystalluria using NanoSight Tracking Analysis. Data are from n = 34–40 healthy participants. *p < 0.05, ****p < 0.0001.

Urine volume, pH, creatinine, calcium, and phosphate levels were all significantly elevated following the oxalate load (**Table 2**). In contrast, both urinary citrate levels and osmolality were significantly decreased (**Table 2**). Both crystalline and soluble urinary oxalate excretion significantly increased after the load (**Figures 2A, B**). The mean concentration of urinary nanocrystals were higher in post-oxalate samples compared to pre-oxalate samples (pre-oxalate 5.8E+07 ± 3.7E+07 vs. post-oxalate 3.8E+08 ± 2.4E+08 particles/ml) (**Figure 2C**).

**Table 2:** Urine chemistries of study participants.

	Pre-Oxalate	Post-Oxalate	p Value
**Urine**			
Volume (ml)	76.1 ± 4.4	863.5 ± 79.1	**0.0000000000027**
pH	6.3 ± 0.1	7.1 ± 0.1	**0.0000000000026**
Creatinine (g)	0.07 ± 0.01	0.23 ± 0.02	**0.0000000033**
Calcium (mg/g Cr)	83.62 ± 9.1	87.73 ± 11.0	0.772
Phosphate (mg/g Cr)	275.99 ± 19.3	266.44 ± 28.1	0.734
Citrate (mg/g Cr)	1805.18 ± 210.7	325.93 ± 41.9	**0.000000026**
Osmolality (mOSM/kg)	414.3 ± 34.2	261.0 ± 25.0	**0.000079**

Values are expressed as mean ± SE.

Bolded p values represent the significant difference between pre-oxalate and post-oxalate samples.

### Dietary Oxalate Loading Impacts Monocyte Cellular Bioenergetics and Mitochondrial Complex Activity

**Figure 3:**
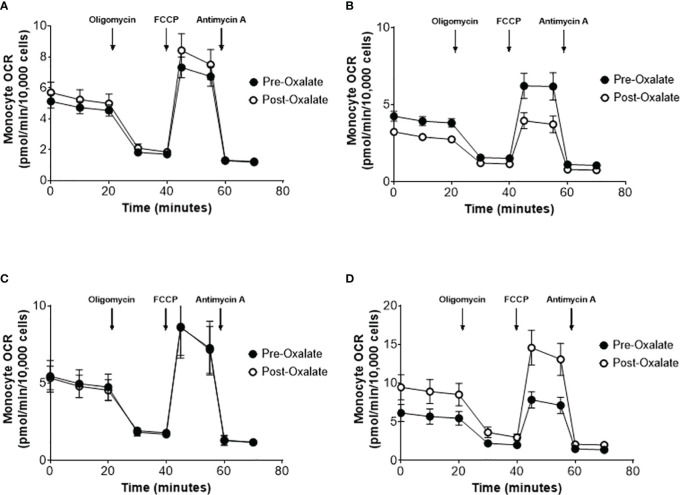
Monocyte cellular bioenergetics varies in healthy participants in response to a dietary oxalate load. Representative oxygen consumption rate (OCR) profiles of **(A)** all study participants pre-oxalate (black circles) and post-oxalate responses (white circles). Participants were further classified as **(B)** decreased, **(C)** same, or **(D)** increased responders based on their post-oxalate responses (white circles). Data are from n = 40 healthy participants and expressed as mean ± SE using technical replicates of 3–5 wells per group.

When combining the responses of all participants, the oxalate load did not cause a significant difference in monocyte cellular bioenergetics (**Figure 3A**). However, there were variable responses in cellular bioenergetics which we categorized based on their oxygen consumption rate (OCR) profiles into three groups: decreased (n = 19, 48%), non-responders (n = 9, 22%), or increased responders (n = 12, 30%). A post-oxalate OCR response of ± 2 SEM was used for this categorization. As shown in **Figure 3B**, the decreased cohort cellular bioenergetic profile was lower in post-oxalate monocytes compared to pre-oxalate monocytes. Specifically, basal respiration, maximal respiration, and reserve capacity were significantly decreased. Nine individuals did not have any changes in their cellular bioenergetic profiles following the load (**Figure 3C**); whereas, 12 individuals had an increased cellular bioenergetic profile in their post-oxalate monocytes compared to their pre-oxalate monocytes (**Figure 3D**). Specifically, they had increased basal, ATP-linked, and maximal respiration and reserve capacity. Mitochondrial complex activity was also measured to identify respiratory complexes impacted by oxalate and was different among the cohort (**Table 3**). Decreased responders had a significant decrease in Complex I, Complex IV, Complex II-FCCP activity, and State 3 respiration. As expected, mitochondrial parameters were not altered in non-responders. In contrast, increased responders had a significant increase in Complex I activity only (**Table 3**). The oxalate free load did not alter cellular bioenergetics in any of the participants (**Supplementary Figure 1**). Individuals classified as decreased responders, when challenged without spinach, became non-responders (**Supplementary Figures 1A, B**). In addition, increased responders, when challenged without spinach, also became non-responders (**Supplementary Figures 1C–E**). **Supplementary Figure 2** shows representative responses from participants categorized in the decreased and increased responders group following consumption of dietary oxalate on two different days.

**Table 3:** Mitochondrial complex activities of study participants (n = 20).

	Pre-Oxalate	Post-Oxalate	*p Value*
**Complex I,** *Decreased*	3.66 ± 0.56	2.84 ± 0.44	**0.009**
*Same*	1.48 ± 0.16	2.00 ± 0.25	0.230
*Increased*	2.71 ± 0.70	4.25 ± 0.81	**0.017**
**Complex IV,** *Decreased*	12.93 ± 2.52	8.55 ± 1.99	**0.034**
*Same*	10.83 ± 0.35	9.54 ± 0.64	0.366
*Increased*	11.74 ± 2.34	12.95 ± 2.03	0.531
**Complex II-ADP,** *Decreased*	4.92 ± 0.79	3.89 ± 0.67	0.07
*Same*	5.28 ± 0.27	3.97 ± 0.25	0.239
*Increased*	3.29 ± 1.09	3.68 ± 0.85	0.502
**Complex II-FCCP,** *Decreased*	7.48 ± 1.07	5.07 ± 0.93	**0.002**
*Same*	4.77 ± 0.24	3.83 ± 0.37	0.380
*Increased*	5.99 ± 2.32	5.75 ± 1.80	0.828
**State 3,** *Decreased*	4.33 ± 0.52	3.51 ± 0.44	**0.026**
*Same*	3.92 ± 0.27	3.30 ± 0.23	0.474
*Increased*	3.27 ± 1.19	3.52 ± 1.33	0.642
**State 4,** *Decreased*	1.35 ± 0.22	1.55 ± 0.30	0.378
*Same*	3.50 ± 0.30	2.30 ± 0.18	0.266
*Increased*	1.15 ± 0.15	1.67 ± 0.47	0.255
**RCR,** *Decreased*	0.23 ± 0.02	0.18 ± 0.02	0.109
*Same*	0.10 ± 0.01	0.12 ± 0.01	0.195
*Increased*	0.21 ± 0.09	0.15 ± 0.05	0.410

—Participants categorized as decreased (n = 9), same (n = 6), or increased responders (n = 5).

—ADP, Adenosine 5′-diphosphate; FCCP, carbonyl cyanide 4-(trifluoromethoxy) phenylhydrazone; RCR, respiratory control ratio.

—Values are expressed as mean ± SE.

Bolded p values represent the significant difference between pre-oxalate and post-oxalate samples.

### Urinary Oxalate Levels and Monocyte Cellular Bioenergetic Responses

Soluble urinary oxalate levels were significantly increased in all three cohorts (**Supplementary Figures 3A–C**). In contrast, individuals from the decreased responder group were the only individuals to have a significant increase in crystalline oxalate levels (**Supplementary Figure 3D**). To evaluate these relationships further, unadjusted relationships between urinary analytes and monocyte cellular bioenergetic parameters were determined (**Table 4**). Soluble oxalate significantly correlated with proton leak (r = 0.392, p = 0.024) and total oxalate (r = 0.691, p < 0.001). To test whether urine analytes could predict monocyte cellular bioenergetic responses, several adjusted predictive regression models for age and gender were tested (**Table 5**). No predictive models were identified between oxalate and the cellular bioenergetic parameters following adjustments. However, proton leak was predictive of citrate (Model p = 0.002, R^2^ = 0.392, p = 0.028) and citrate was predictive of proton leak (Model p = 0.064, R^2^ = 0.225, p = 0.028).

**Table 4:** Unadjusted correlations between monocyte cellular energetic responses and urinary analyte levels.

	Basal	ATP-Linked	Proton-Leak	Maximal	Reserve Capacity	Total Oxalate	Soluble Oxalate	Crystalline Oxalate	Calcium	Citrate
**Basal**										
**ATP-Linked**	**r = 0.992** **p < 0.001** (n = 40)									
**Proton-Leak**	**r = 0.789** **p < 0.001** (n = 40)	**r = 0.710** **p < 0.001** (n = 40)								
**Maximal**	**r = 0.766** **p < 0.001** (n = 40)	**r = 0.794** **p < 0.001** (n = 40)	**r = 0.454** **p = 0.003** (n = 40)							
**Reserve Capacity**	**r = 0.508** **p < 0.001** (n = 40)	**r = 0.550** **p < 0.001** (n = 40)	r = 0.197 p = 0.22 (n = 40)	**r = 0.940** **p < 0.001** (n = 40)						
**Total Oxalate**	r = 0.001 p = 0.99 (n = 33)	r = -0.021 p = 0.91 (n = 33)	r = 0.105 p = 0.56 (n = 33)	r = -0.008 p = 0.96 (n = 33)	r = -0.020 p = 0.91 (n = 33)					
**Soluble Oxalate**	r = 0.332 p = 0.059 (n = 33)	r = 0.305 p = 0.085 (n = 33)	**r = 0.392** **p = 0.024** (n = 33)	r = 0.139 p = 0.44 (n = 33)	r = 0.010 p = 0.96 (n = 33)	**r = 0.691** **p < 0.001** (n = 33)				
**Crystalline Oxalate**	r = 0.149 p = 0.39 (n = 35)	r = 0.146 p = 0.40 (n = 35)	r = 0.004 p = 0.98 (n = 35)	r = 0.164 p = 0.35 (n = 35)	r = 0.140 p = 0.42 (n = 35)	r = -0.032 p = 0.86 (n = 32)	r = -0.050 p = 0.78 (n = 32)			
**Calcium**	r = 0.161 p = 0.43 (n = 26)	r = 0.156 p = 0.45 (n = 26)	r = 0.098 p = 0.64 (n = 26)	r = 0.179 p = 0.38 (n = 26)	r = 0.152 p = 0.46 (n = 26)	r = 0.028 p = 0.90 (n = 21)	r = 0.130 p = 0.58 (n = 21)	r = 0.385 p = 0.070 (n = 23)		
**Citrate**	r = 0.161 p = 0.33 (n = 39)	r = 0.132 p = 0.42 (n = 39)	r = 0.241 p = 0.14 (n = 39)	r = 0.077 p = 0.64 (n = 39)	r = 0.029 p = 0.86 (n = 39)	r = -0.281 p = 0.12 (n = 32)	r = -0.214 p = 0.24 (n = 32)	r = -0.013 p = 0.56 (n = 34)	r = 0.314 p = 0.12 (n = 26)	

Bolded values also represent significant difference between data points shown in **Tables 4** and **5**.

**Table 5:** Adjusted predictive regression models of monocyte cellular bioenergetic responses and urinary analyte levels.

	Basal	ATP-Linked	Proton-Leak	Maximal	Reserve Capacity	Total Oxalate	Soluble Oxalate	Crystalline Oxalate	Calcium	Citrate
**Basal**	Model p = 0.15 R2 = 0.137 (n = 40)	**Model p < 0.001** **R2 = 0.984** **p < 0.001** **(n = 40)**	**Model p < 0.001** **R2 = 0.635** **p < 0.001** **(n = 40)**	**Model p < 0.001** **R2 = 0.598** **p < 0.001** **(n = 40)**	**Model p = 0.022** **R2 = 0.274** **p = 0.002** **(n = 40)**	Model p = 0.99 R2 = 0.010 p = 0.88 (n = 33)	Model p = 0.46 R2 = 0.117 p = 0.17	Model p = 0.078 R2 = 0.238 p = 0.14 (n = 35)	Model p = 0.44 R2 = 0.158 p = 0.29 (n = 26)	Model p = 0.009 R2 = 0.323 p = 0.26 (n = 39)
**ATP-Linked**	**Model p < 0.001** **R2 = 0.984** **p < 0.001** **(n = 40)**	Model p = 0.18 R2 = 0.125 (n = 40)	**Model p < 0.001** **R2 = 0.523** **p < 0.001** **(n = 40)**	**Model p < 0.001** **R2 = 0.637** **p < 0.001** **(n = 40)**	**Model p = 0.009** **R2 = 0.316** **p < 0.001** **(n = 40)**	Model p = 0.99 R2 = 0.012 p = 0.78 (n = 33)	Model p = 0.53 R2 = 0.104 p = 0.23 (n = 33)	Model p = 0.087 R2 = 0.231 p = 0.17 (n = 35)	Model p = 0.45 R2 = 0.155 p = 0.30 (n = 26)	Model p = 0.011 R2 = 0.311 p = 0.41 (n = 39)
**Proton-Leak**	**Model p < 0.001** **R2 = 0.648** **p < 0.001** **(n = 40)**	**Model p < 0.001** **R2 = 0.534** **p < 0.001** **(n = 40)**	Model p = 0.26 R2 = 0.105 (n = 40)	**Model p = 0.049** **R2 = 0.233** **p = 0.008** **(n = 40)**	Model p = 0.67 R2 = 0.064 P = 0.29 (n = 40)	Model p = 0.97 R2 = 0.019 p = 0.60 (n = 33)	Model p = 0.30 R2 = 0.156 p = 0.077 (n = 33)	Model p = 0.17 R2 = 0.186 p = 0.64 (n = 35)	Model p = 0.54 R2 = 0.132 p = 0.47 (n = 26)	**Model p = 0.002** **R2 = 0.392** **p = 0.028** **(n = 39)**
**Maximal**	**Model p < 0.001** **R2 = 0.630** **p < 0.001** **(n = 40)**	**Model p < 0.001** **R2 = 0.662** **p < 0.001** **(n = 40)**	**Model p = 0.024** **R2 = 0.268** **p = 0.008** **(n = 40)**	Model p = 0.50 R2 = 0.062 (n = 40)	**Model p < 0.001** **R2 = 0.892** **P < 0.001** **(n = 40)**	Model p = 0.99 R2 = 0.010 p = 0.88 (n = 33)	Model p = 0.76 R2 = 0.062 p = 0.64 (n = 33)	Model p = 0.11 R2 = 0.218 p = 0.23 (n = 35)	Model p = 0.51 R2 = 0.139 p = 0.42 (n = 26)	Model p = 0.015 R2 = 0.299 p = 0.77 (n = 39)
**Reserve Capacity**	**Model p = 0.004** **R2 = 0.352** **p = 0.002** **(n = 40)**	**Model p = 0.002** **R2 = 0.381** **p < 0.001** **(n = 40)**	Model p = 0.27 R2 = 0.133 P = 0.29 (n = 40)	**Model p < 0.001** **R2 = 0.895** **P < 0.001** **(n = 40)**	Model p = 0.74 R2 = 0.034 (n = 40)	Model p = 0.99 R2 = 0.010 p = 0.85 (n = 33)	Model p = 0.80 R2 = 0.055 p = 0.95 (n = 33)	Model p = 0.14 R2 = 0.202 p = 0.37 (n = 35)	Model p = 0.58 R2 = 0.123 p = 0.59 (n = 26)	Model p = 0.015 R2 = 0.297 p = 0.90 (n = 39)
**Total Oxalate**	Model p = 0.046 R2 = 0.285 p = 0.88 (n = 33)	Model p = 0.073 R2 = 0.256 p = 0.78 (n = 33)	Model p = 0.078 R2 = 0.252 p = 0.60 (n = 33)	Model p = 0.63 R2 = 0.086 p = 0.88 (n = 33)	Model p = 0.90 R2 = 0.037 p = 0.85 (n = 33)	Model p = 0.97 R2 = 0.009 (n = 33)	**Model p < 0.001** **R2 = 0.523** **P < 0.001** **(n = 33)**	Model p = 0.29 R2 = 0.163 p = 0.81 (n = 32)	Model p = 0.84 R2 = 0.080 p = 0.94 (n = 21)	Model p = 0.008 R2 = 0.393 p = 0.095 (n = 32)
**Soluble Oxalate**	Model p = 0.020 R2 = 0.331 p = 0.17 (n = 33)	Model p = 0.040 R2 = 0.293 p = 0.23 (n = 33)	Model p = 0.022 R2 = 0.336 p = 0.077 (n = 33)	Model p = 0.59 R2 = 0.092 p = 0.64 (n = 33)	Model p = 0.90 R2 = 0.036 p = 0.95 (n = 33)	**Model p < 0.001** **R2 = 0.500** **p < 0.001** **(n = 33)**	Model p = 0.65 R2 = 0.055 (n = 33)	Model p = 0.29 R2 = 0.163 p = 0.78 (n = 32)	Model p = 0.74 R2 = 0.110 p = 0.48 (n = 21)	Model p = 0.009 R2 = 0.383 p = 0.12 (n = 32)
**Crystalline Oxalate**	Model p = 0.081 R2 = 0.236 p = 0.14 (n = 35)	Model p = 0.12 R2 = 0.208 p = 0.17 (n = 35)	Model p = 0.18 R2 = 0.186 p = 0.64 (n = 35)	Model p = 0.46 R2 = 0.111 p = 0.23 (n = 35)	Model p = 0.76 R2 = 0.059 p = 0.37 (n = 35)	Model p = 0.99 R2 = 0.009 p = 0.81 (n = 33)	Model p = 0.72 R2 = 0.072 p = 0.78 (n = 32)	Model p = 0.10 R2 = 0.179 (n = 35)	Model p = 0.35 R2 = 0.209 p = 0.11 (n = 23)	Model p = 0.018 R2 = 0.329 p = 0.70 (n = 34)
**Calcium**	Model p = 0.47 R2 = 0.150 p = 0.29 (n = 26)	Model p = 0.47 R2 = 0.149 p = 0.30 (n = 26)	Model p = 0.86 R2 = 0.057 p = 0.47 (n = 26)	Model p = 0.65 R2 = 0.107 p = 0.42 (n = 26)	Model p = 0.78 R2 = 0.078 p = 0.59 (n = 26)	Model p = 0.99 R2 = 0.014 p = 0.94 (n = 21)	Model p = 0.51 R2 = 0.175 p = 0.48 (n = 21)	Model p = 0.15 R2 = 0.298 p = 0.11 (n = 23)	Model p = 0.45 R2 = 0.111 (n = 26)	Model p = 0.20 R2 = 0.239 p = 0.29 (n = 26)
**Citrate**	Model p = 0.15 R2 = 0.177 p = 0.26 (n = 39)	Model p = 0.21 R2 = 0.152 p = 0.41 (n = 39)	**Model p = 0.064** **R2 = 0.225** **p = 0.028** **(n = 39)**	Model p = 0.60 R2 = 0.076 p = 0.77 (n = 39)	Model p = 0.81 R2 = 0.045 p = 0.90 (n = 39)	Model p = 0.52 R2 = 0.108 p = 0.095 (n = 32)	Model p = 0.39 R2 = 0.137 p = 0.12 (n = 32)	Model p = 0.27 R2 = 0.157 p = 0.70 (n = 34)	Model p = 0.44 R2 = 0.157 p = 0.29 (n = 26)	Model p = 0.006 R2 = 0.300 (n = 39)

The columns represent the variables when they are used as dependent variables, while the rows represent the variables when they are used as independent predictor variables. All models contain the independent predictor variable; age, race, and gender as covariates; and the dependent variable. The first entry in each table cell is the model R2 value (goodness of fit); the second entry is the p-value for the independent predictor variable; and the final entry is the number of observations included in the model. Results for models containing statistically significant (p < 0.05) predictor variables are bolded. The diagonal entries describe models that were run with only the covariates present (i.e., an independent predictor variable was not included in these models).

### Dietary Oxalate Alters Monocyte Anti-Inflammatory and Pro-Inflammatory Signaling

**Figure 4:**
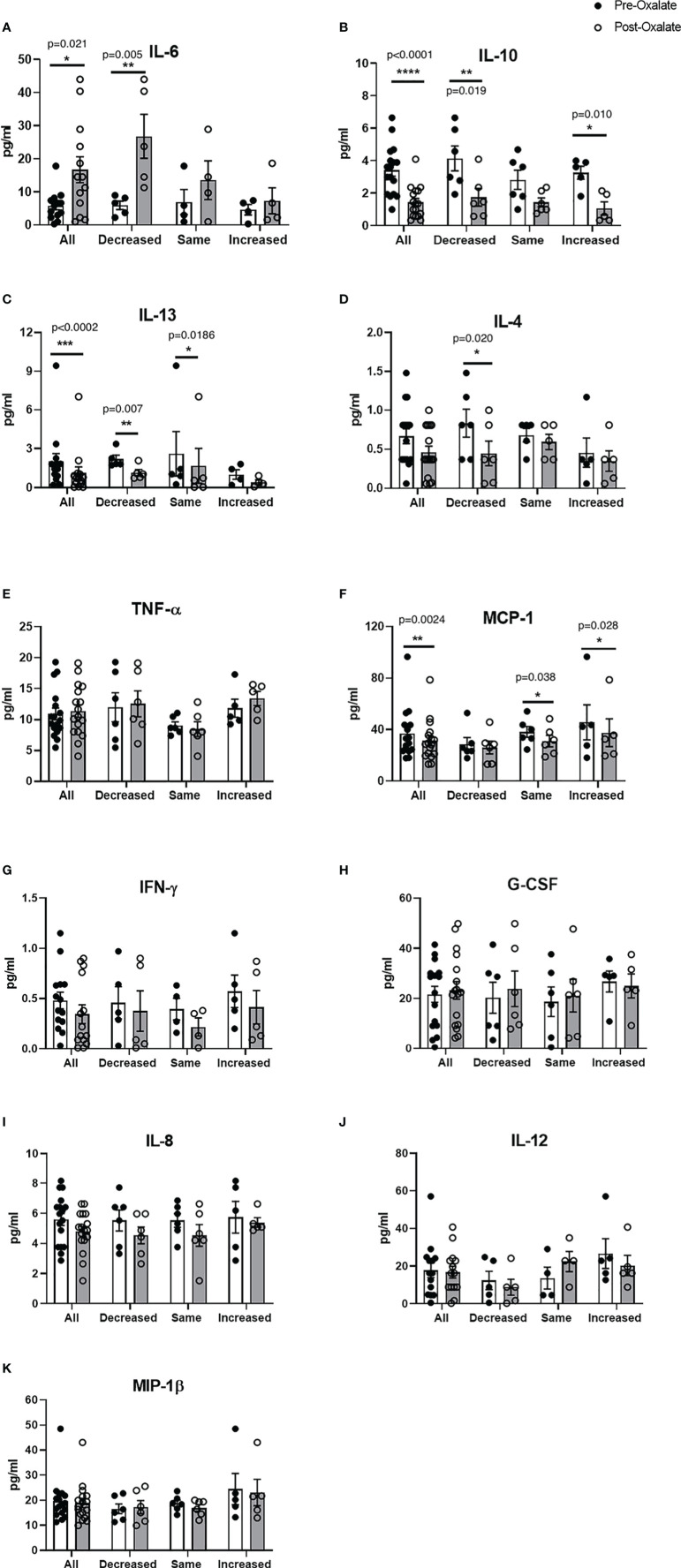
Plasma cytokine and chemokine levels vary in healthy participants in response to a dietary oxalate load. Multiplex analysis of cytokines **(A)** IL-6, **(B)** IL-10, **(C)** IL-13, **(D)** IL-4, **(E)** TNF-ɑ, **(F)** MCP-1, **(G)** IFN-γ, **(H)** G-CSF, **(I)** IL-8, **(J)** IL-12, and **(K)** MIP-1β in pre-oxalate and post-oxalate plasma samples from healthy participants. Data are from n = 18 healthy participants. *p < 0.05, **p < 0.01, ***p < 0.001, ****p < 0.0001.

We further studied plasma cytokine and chemokine levels in healthy participants before and after the oxalate load. Collectively, oxalate caused a significant increase in pro-inflammatory IL-6 levels (**Figure 4A**) and significantly reduced IL-10 (anti-inflammatory cytokine, **Figure 4B**), IL-13 (anti-inflammatory cytokines, **Figure 4C**), and MCP-1 (monocyte recruitment, **Figure 4F**) levels. Oxalate had no effect on IL-4, TNF-ɑ, IFN-γ, G-CSF, IL-8, IL-12, and MIP-1β (**Figures 4D, E, G–K**). However, oxalate did cause differential responses in plasma cytokine and chemokine levels within the monocyte cellular bioenergetic responders. The decreased cellular bioenergetic cohort had a significant increase in IL-6 levels (**Figure 4A**) and a significant decrease in IL-10, IL-13, and IL-4 (all anti-inflammatory cytokines) in their post-oxalate samples compared to pre-oxalate samples (**Figures 4B–D**). Individuals classified as same responders had a significant decrease only in IL-13 (**Figure 4C**) and MCP-1 (**Figure 4F**) levels. Increased responders had a significant decrease in IL-10 (**Figure 4B**) and MCP-1 levels (**Figure 4F**) in their post-oxalate plasma compared to their pre-oxalate plasma samples. Additional cytokines (i.e., IL-1β, IL-2, IL-5, IL-7, IL-17A, and GM-CSF) were not detected in any of the samples.

## Discussion

This study aimed to determine whether a dietary oxalate mediated increase in urinary oxalate excretion and crystalluria modifies monocyte cellular bioenergetics, and immune responses in humans. To achieve this, healthy adults consumed low oxalate diets, prior to drinking a blended preparation of fruits and vegetables containing a large amount of oxalate, equivalent to the amount found in a spinach salad. This underscores the “real world” approach that we undertook in this study. We have previously reported that sodium oxalate loads increase urinary oxalate levels in humans (13). Consistent with these findings, we show consumption of a blended liquid preparation with oxalate levels that individuals could consume in daily life, substantially increases urinary oxalate excretion and nanocrystal formation. Urinary crystals are proposed to be a reliable predicator of stone formation and recurrence in CaOx stone formers (38). Smaller crystals (nm range) have been suggested to cause more injury to renal cells compared to larger crystals (39). We previously reported that CaOx crystals suppress cellular bioenergetics and disturb redox homeostasis in monocytes (31). Additionally, CaOx crystals have been shown to differentiate human monocytes into pro-inflammatory macrophages (40).

In the current study, we show that a dietary oxalate load impacts circulating monocytes. We demonstrated that there were three types of cellular bioenergetic responses to a dietary oxalate load in healthy adults. This was expected since human participants have different genetic backgrounds, immune systems, and environmental exposures. Of the forty participants, almost fifty percent of the individuals had decreased cellular bioenergetic profiles which was comparable to previously tested CaOx stone formers (30). In additional experiments, we determined that a subset of individuals who also consumed a load containing “no oxalate” did not have any changes in their monocyte cellular bioenergetics, suggesting that the responses observed were likely due to oxalate or perhaps from other components of spinach. These individuals were from both decreased and increased cohorts. To further delineate the mechanisms leading to these varied bioenergetic responses, mitochondrial complex activities were measured. Complex I, Complex IV, and Complex II-FCCP activities were significantly reduced in the decreased responder group. A reduction in mitochondrial complex activity could lead to reduced ATP levels and increased ROS generation, inflammation, and cell death (41, 42). As expected, mitochondrial complex activity was not altered in the non-responder group. In contrast, complex I activity was significantly elevated in the increased responder group. This suggests oxalate could be targeting monocyte mitochondrial complexes and could lead to changes in monocyte cellular bioenergetics.

We undertook modeling to assess for any relationships between oxalate excretion and monocyte responses. We constructed predictive models from the initial 20 participants and tested the model in the last 20 participants who completed the study. The unadjusted analyses showed urinary soluble oxalate levels correlated with proton leak. However, this was lost when we adjusted for age and gender. We did determine that proton leak was predictive of citrate excretion and vice versa. This is not unexpected as the citric acid cycle occurs in the mitochondria. We expect testing additional models could be useful to predict responses in a larger cohort of healthy individuals and patients for future studies.

Several cytokines including IL-6, TNF-ɑ, IL-1β, have been reported to be influenced by diet (43). To determine whether dietary oxalate loading could modify cytokine and chemokine levels in circulation, we measured plasma cytokines and chemokine levels using a multiplex immunoassay system. We previously reported that patients with CaOx kidney stones have elevated plasma IL-6 cytokine levels (30). Consistent with these findings, we determined that a dietary oxalate load significantly increased IL-6 levels in healthy adults. Furthermore, oxalate significantly reduced key anti-inflammatory cytokines (i.e., IL-10 and IL-13) and a chemokine (i.e., MCP-1) vital for monocyte recruitment. Oxalate also caused differential inflammatory signaling in the three different types of monocyte cellular bioenergetic responders. The decreased responders had a significant increase in IL-6 plasma levels which is consistent with our earlier data in CaOx stone formers (30). These same individuals also had decreased anti-inflammatory cytokine (i.e., IL-13, IL-10, and IL-4) levels. There was no change in IL-6 levels in the same or increased cohort. Further, we did not observe any correlation between plasma cytokines and chemokines with monocyte cellular bioenergetics. These data support the premise that a dietary oxalate load promotes pro-inflammatory responses and reduces anti-inflammatory responses and this could be occurring in the patient population.

This study demonstrated that dietary oxalate loading increases urinary oxalate excretion and nanocrystalluria. Notably, the majority of our study participants had increased urinary oxalate excretion after the load presumably due to increased gastrointestinal oxalate absorption. The increased inflammation and reduced monocyte cellular bioenergetics in the decreased responders can’t be directly attributable to nanocrystals as they have not been shown to be present in circulating blood. It is conceivable that both nanocrystalluria and increased delivery of oxalate to the kidney could generate changes in the renal environment which could alter macrophage function and generate inflammation. The changes seen in the circulating monocytes in the decreased responders may be due to increased oxalate exposure as plasma oxalate levels have been shown to increase after oral sodium oxalate loading (13). Some of these monocytes are delivered to the kidney and differentiated into macrophages with potentially altered cellular bioenergetics which may play a role in kidney stone formation in susceptible individuals. The non-responders and increased responders may have protective mechanisms which prevent such outcomes.

Some of the limitations of this study include the small sample size which drove us to perform multiple paired t-tests which could have introduced type-1 error. Twenty-four hour urine samples were not collected since the study was designed to determine the effects of a dietary oxalate load on immune cell function at 5 h. This time point was selected because we previously determined that urinary oxalate levels peak at this time following a sodium oxalate load (13). Another limitation is that plasma oxalate levels were not recorded which could have influenced response. When a subset of participants received a load without spinach, these responses were not reproduced. It is possible that some other component of spinach could be driving this response.

Future studies will include a larger cohort of healthy individuals and calcium oxalate kidney stone formers to better delineate mechanisms influencing monocyte metabolism after an oxalate load and to test additional predictive models. *In vitro* studies will also be used to further dissect the pro-inflammatory and pro-oxidative signaling pathways involved and their direct impact on cellular bioenergetics. Understanding such mechanisms could aid in identifying targets to improve monocyte metabolism and immune responses as this may be associated with kidney stone risk. In conclusion, these findings demonstrate that a dietary oxalate load influences monocyte metabolism and immune function.

## Data Availability Statement

The datasets presented in this article are not readily available because study participants were assured raw data would remain confidential and would not be shared. Requests to access the datasets should be directed to TM, taneciamitchell@uabmc.edu.

## Ethics Statement

The studies involving human participants were reviewed and approved by The University of Alabama at Birmingham Institutional Review Board. The patients/participants provided their written informed consent to participate in this study.

## Author Contributions

DA, RO, and TM designed research studies. MP, DA, and TM acquired informed consent. PK, MP, VY, AA, and TM conducted experiments. PK, MP, VY, AA, and TM acquired data. PK, MP, RO, VY, AA, and TM analyzed data. PK, MP, RO, DA, and TM wrote the manuscript. TM supervised the study. All authors contributed to the article and approved the submitted version.

## Funding

Research reported in this publication was supported by NIH grants DK106284 and DK123542 (TM), UL1TR003096, and P30 DK079337; and the UAB Faculty Development Grant Program at UAB (TM).

## Conflict of Interest

The authors declare that the research was conducted in the absence of any commercial or financial relationships that could be construed as a potential conflict of interest.
