# Supplementary material for: Dietary Oxalate Loading Impacts Monocyte Metabolism and Inflammatory Signaling in Humans
Source: Front Immunol. 2021 Feb 25;12:617508. doi: 10.3389/fimmu.2021.617508 (PMC7959803; doi:10.3389/fimmu.2021.617508)
Supplement: Supplementary file 1 [file Presentation_1.pptx]

## Slide 1
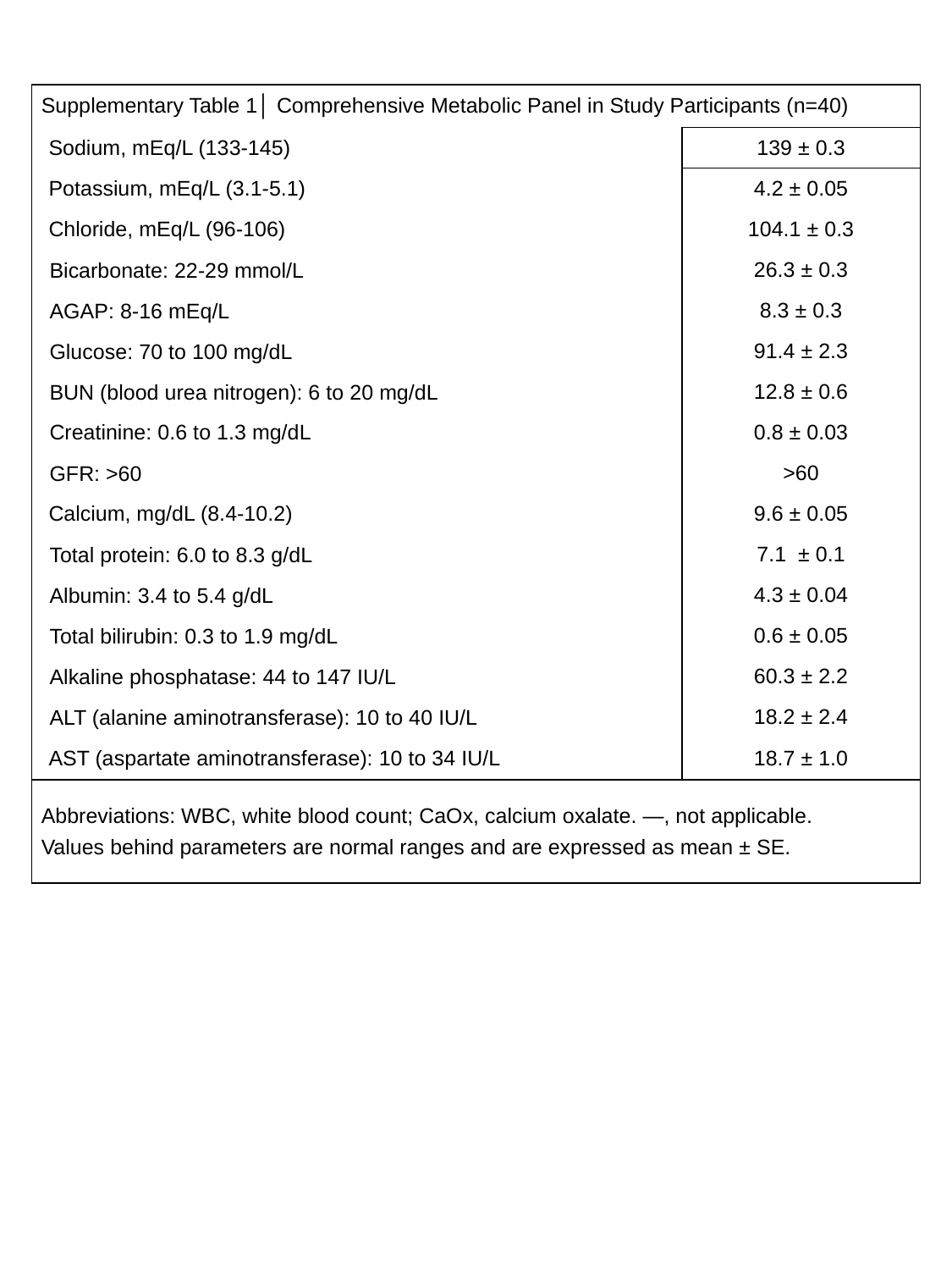

| Supplementary Table 1│ Comprehensive Metabolic Panel in Study Participants (n=40) | |
| --- | --- |
| Sodium, mEq/L (133-145) | 139 ± 0.3 |
| Potassium, mEq/L (3.1-5.1) | 4.2 ± 0.05 |
| Chloride, mEq/L (96-106) | 104.1 ± 0.3 |
| Bicarbonate: 22-29 mmol/L | 26.3 ± 0.3 |
| AGAP: 8-16 mEq/L | 8.3 ± 0.3 |
| Glucose: 70 to 100 mg/dL | 91.4 ± 2.3 |
| BUN (blood urea nitrogen): 6 to 20 mg/dL | 12.8 ± 0.6 |
| Creatinine: 0.6 to 1.3 mg/dL | 0.8 ± 0.03 |
| GFR: >60 | >60 |
| Calcium, mg/dL (8.4-10.2) | 9.6 ± 0.05 |
| Total protein: 6.0 to 8.3 g/dL | 7.1 ± 0.1 |
| Albumin: 3.4 to 5.4 g/dL | 4.3 ± 0.04 |
| Total bilirubin: 0.3 to 1.9 mg/dL | 0.6 ± 0.05 |
| Alkaline phosphatase: 44 to 147 IU/L | 60.3 ± 2.2 |
| ALT (alanine aminotransferase): 10 to 40 IU/L | 18.2 ± 2.4 |
| AST (aspartate aminotransferase): 10 to 34 IU/L | 18.7 ± 1.0 |
| Abbreviations: WBC, white blood count; CaOx, calcium oxalate. —, not applicable. Values behind parameters are normal ranges and are expressed as mean ± SE. | |

## Slide 2
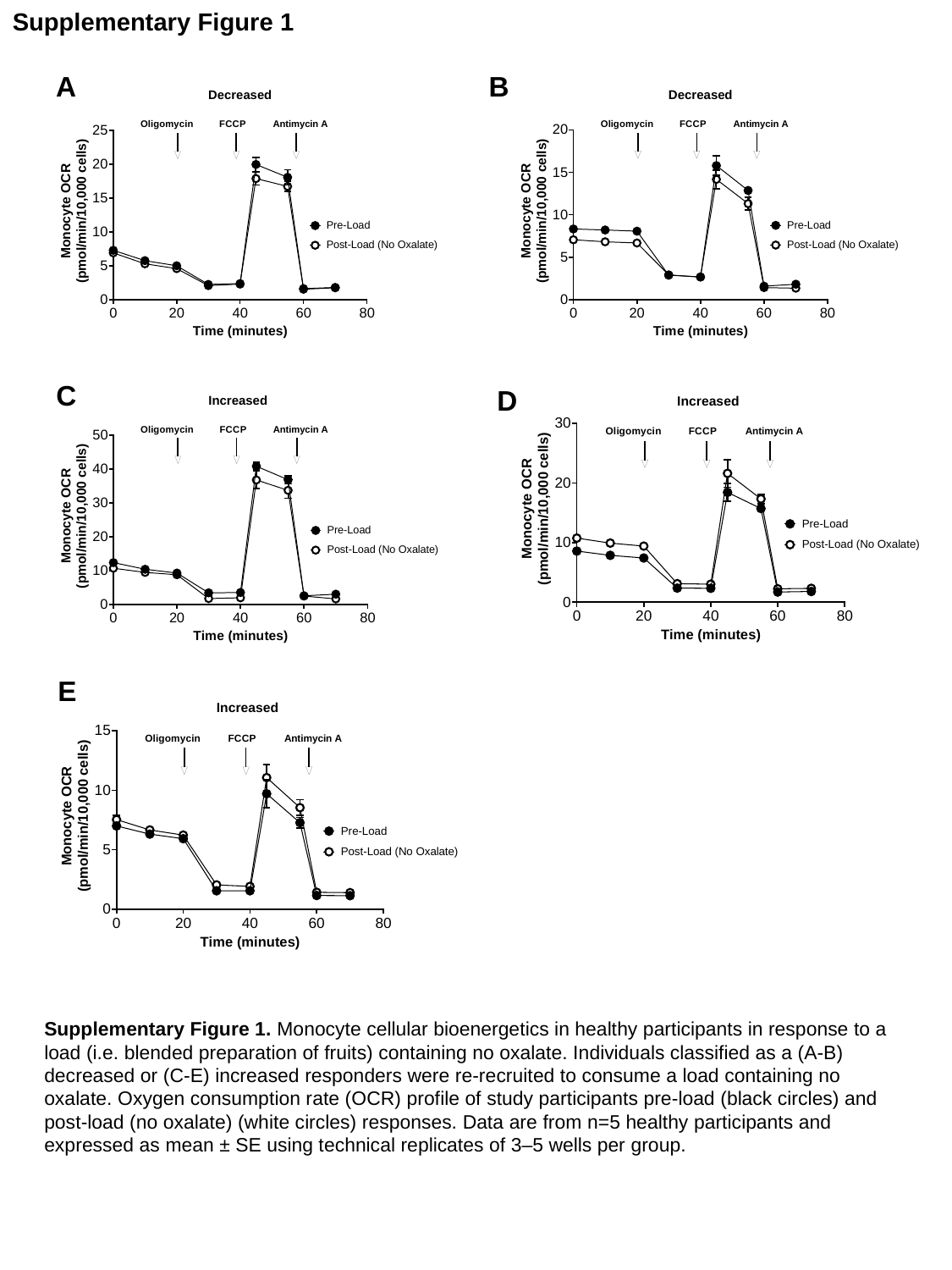

Supplementary Figure 1
A
B
C
D
E
Supplementary Figure 1. Monocyte cellular bioenergetics in healthy participants in response to a load (i.e. blended preparation of fruits) containing no oxalate. Individuals classified as a (A-B) decreased or (C-E) increased responders were re-recruited to consume a load containing no oxalate. Oxygen consumption rate (OCR) profile of study participants pre-load (black circles) and post-load (no oxalate) (white circles) responses. Data are from n=5 healthy participants and expressed as mean ± SE using technical replicates of 3–5 wells per group.

## Slide 3
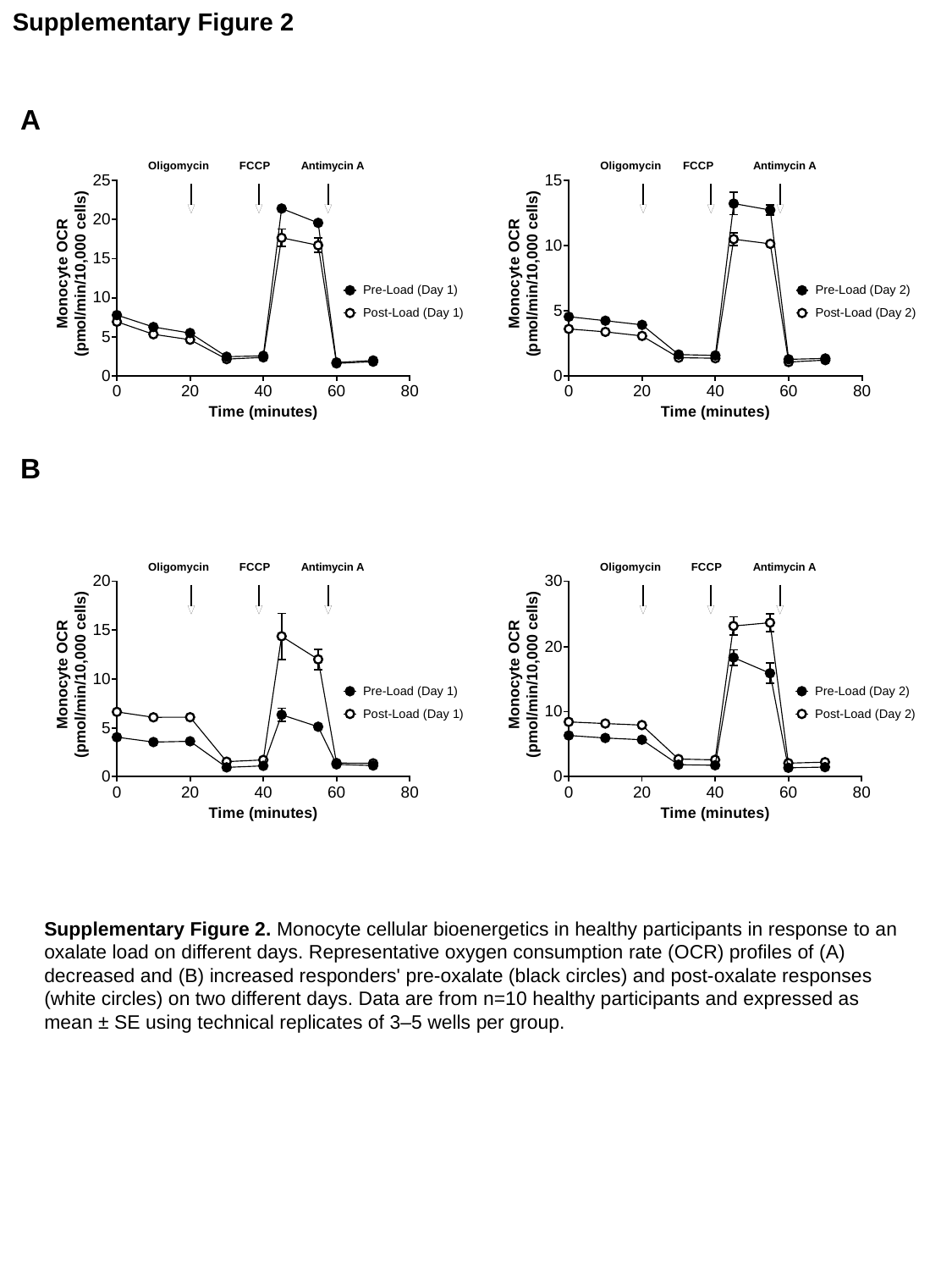

Supplementary Figure 2
A
B
Supplementary Figure 2. Monocyte cellular bioenergetics in healthy participants in response to an oxalate load on different days. Representative oxygen consumption rate (OCR) profiles of (A) decreased and (B) increased responders' pre-oxalate (black circles) and post-oxalate responses (white circles) on two different days. Data are from n=10 healthy participants and expressed as mean ± SE using technical replicates of 3–5 wells per group.

## Slide 4
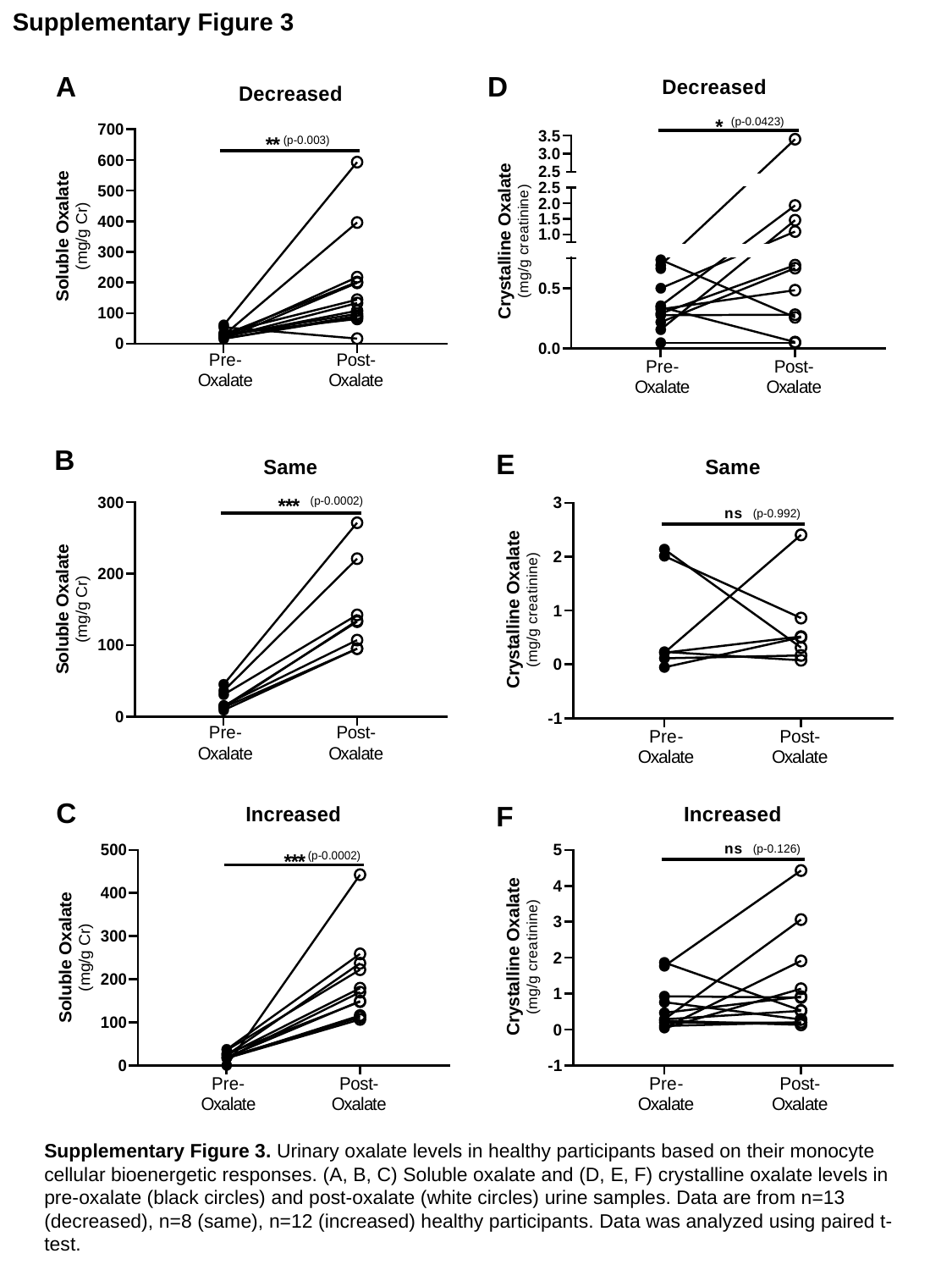

Supplementary Figure 3
D
A
B
E
C
F
Supplementary Figure 3. Urinary oxalate levels in healthy participants based on their monocyte cellular bioenergetic responses. (A, B, C) Soluble oxalate and (D, E, F) crystalline oxalate levels in pre-oxalate (black circles) and post-oxalate (white circles) urine samples. Data are from n=13 (decreased), n=8 (same), n=12 (increased) healthy participants. Data was analyzed using paired t-test.
